# Neuronal excitation upregulates *Tbr1*, a high-confidence risk gene of autism, mediating *Grin2b* expression in the adult brain

**DOI:** 10.3389/fncel.2014.00280

**Published:** 2014-09-10

**Authors:** Hsiu-Chun Chuang, Tzyy-Nan Huang, Yi-Ping Hsueh

**Affiliations:** ^1^Graduate Institute of Life Sciences, National Defense Medical Center, TaipeiTaiwan; ^2^Institute of Molecular Biology, Academia Sinica, TaipeiTaiwan

**Keywords:** autism, *Grin2b*, immediate early gene, neuronal activation, *Tbr1*

## Abstract

The activity-regulated gene expression of transcription factors is required for neural plasticity and function in response to neuronal stimulation. T-brain-1 (TBR1), a critical neuron-specific transcription factor for forebrain development, has been recognized as a high-confidence risk gene for autism spectrum disorders. Here, we show that in addition to its role in brain development, *Tbr1* responds to neuronal activation and further modulates the *Grin2b* expression in adult brains and mature neurons. The expression levels of *Tbr1* were investigated using both immunostaining and quantitative reverse transcription polymerase chain reaction (RT-PCR) analyses. We found that the mRNA and protein expression levels of *Tbr1* are induced by excitatory synaptic transmission driven by bicuculline or glutamate treatment in cultured mature neurons. The upregulation of *Tbr1* expression requires the activation of both α-amino-3-hydroxy-5-methyl-4-isoxazole-propionic acid (AMPA) and *N*-methyl-D-aspartate (NMDA) receptors. Furthermore, behavioral training triggers *Tbr1* induction in the adult mouse brain. The elevation of *Tbr1* expression is associated with *Grin2b* upregulation in both mature neurons and adult brains. Using *Tbr1*-deficient neurons, we further demonstrated that TBR1 is required for the induction of *Grin2b* upon neuronal activation. Taken together with the previous studies showing that TBR1 binds the *Grin2b* promoter and controls expression of luciferase reporter driven by *Grin2b* promoter, the evidence suggests that TBR1 directly controls *Grin2b* expression in mature neurons. We also found that the addition of the calcium/calmodulin-dependent protein kinase II (CaMKII) antagonist KN-93, but not the calcium-dependent phosphatase calcineurin antagonist cyclosporin A, to cultured mature neurons noticeably inhibited *Tbr1* induction, indicating that neuronal activation upregulates *Tbr1* expression in a CaMKII-dependent manner. In conclusion, our study suggests that *Tbr1* plays an important role in adult mouse brains in response to neuronal activation to modulate the activity-regulated gene transcription required for neural plasticity.

## INTRODUCTION

Neuronal activation regulates the activity of transcription factors to modulate the gene expression required for the formation of mature synapses and neural circuits and development of cognitive function (Parkes and Westbrook, 2011). Abnormalities in activity-regulated transcriptional pathways have been implicated in neurodevelopmental disorders (Pfeiffer et al., 2010; King et al., 2013), especially in autism spectrum disorders (ASDs), which are characterized by deficits in social interaction and communication, cognitive inflexibility, repetitive behaviors, and intellectual disability. Recent genetic studies have found recurrent mutations of T-brain-1 (TBR1), encoding a brain-specific T-box transcription factor (Bulfone et al., 1995), in ASD patients and identified *Tbr1* as a causative gene in ASDs (Neale et al., 2012; O’Roak et al., 2012a; Huang et al., 2014).

*Tbr1* is specifically expressed in the olfactory bulb, cerebral cortex, hippocampus, and amygdala (Bulfone et al., 1995, 1998; Medina et al., 2004; Remedios et al., 2007). Its peak expression is observed during the embryonic stage, and the expression gradually decreases after birth (Bulfone et al., 1995). TBR1 protein is expressed at low, but significant, levels in the adult forebrain (Hsueh et al., 2000). At the embryonic stage, *Tbr1*, which is abundantly expressed in post-mitotic projection neurons, controls the axonal connections between the cerebral cortex and thalamus and the formation of the corticospinal tract (Hevner et al., 2001). Studies using knockout mice demonstrated that *Tbr1* plays important roles in the maintenance of neuronal projections in the olfactory bulb (Bulfone et al., 1998), layer and regional identification of the neocortex (Hevner et al., 2001; Bedogni et al., 2010), and migration of the amygdala (Remedios et al., 2007). Moreover, we recently showed that TBR1 modulates expression of *Ntng1*, *Cdh8*, and *Cntn2* and regulates the axonal outgrowth of amygdalar neurons, which are critical for the inter- and intra-amygdalar connections (Huang et al., 2014). All of these studies indicate that *Tbr1* is significantly involved in brain development.

Our previous studies showed that TBR1 interacts with CASK (calcium/calmodulin-dependent serine protein kinase; Hsueh et al., 2000; Hsueh, 2009). Mutations in the *CASK* gene result in X-linked mental retardation (Najm et al., 2008; Tarpey et al., 2009; Moog et al., 2011). The CASK-TBR1 protein complex regulates the expression of *glutamate receptor, ionotropic, N-methyl-D-aspartate 2B* (*Grin2b*, also known as *Nmdar2b*; Wang et al., 2004a,b), which is critical for learning and memory and involved in autism and schizophrenia (Kristiansen et al., 2010; O’Roak et al., 2012b). Recently, we demonstrated that *Tbr1*-deficient mice exhibit autistic-like behaviors (Huang et al., 2014). Upon behavioral stimulation, the induction of *Grin2b* expression was impaired in *Tbr1* heterozygous mice, indicating that *Tbr1* is likely required for the induction of *Grin2b* upon neuronal activation. Thus, in addition to regulating neuronal development, *Tbr1* likely responds to neuronal activation and controls *Grin2b* expression in adult brains.

In literatures, neuronal activation-induced immediate early gene (IEG) responses have been demonstrated to be relevant to dysfunction of glutamatergic pathways and psychological stress. First of all, stress-induced dysregulation in glutamate transmission contributes to development of schizophrenia and other mood disorders (de Kloet et al., 2005; Popoli et al., 2012; Weickert et al., 2013), indicating glutamatergic system has a strong impact on psychiatric disorders. Moreover, acute stress elevates the glucocorticoid levels and activates glucocorticoid receptor, a ligand-inducible nuclear transcription factor, to induce an increase of presynaptic SNARE complexes and thus further enhance glutamate release (Popoli et al., 2012). At the post-synaptic sites, acute stressful conditions induce expression of a series of IEGs, including early growth response 1 (*Egr1*) and activity-regulated cytoskeletal-associated protein (*Arc*), and change α-amino-3-hydroxy-5-methyl-4-isoxazole-propionic acid (AMPA)/*N*-methyl-D-aspartate (NMDA) receptor-mediated glutamatergic transmission. It then impacts on neuronal activation-dependent synaptic plasticity (Revest et al., 2005; Fumagalli et al., 2011; Benekareddy et al., 2013). Besides, both acute behavioral stress in live animals and corticosterone treatment in brain slices increase the synaptic expressions and function of NMDA and AMPA receptors through the upregulation of serum- and glucocorticoid-inducible kinase (SGK), another IEG, and activation of RAB4, a regulator for endocytic recycling of receptors (Yuen et al., 2011). All of these studies support that psychological stress influences the activity-mediated glutamatergic transmission and activates or regulates the downstream genes via IEGs. Since *Tbr1* has been shown to directly regulate the expression of *Grin2b* and *Tbr1* plays roles in psychiatric disorders, *Tbr1* is a likely candidate that senses the neuronal stimulations and regulates glutamatergic pathways.

In this report, we demonstrate that neuronal activation in mature cultured neurons and behavioral stimulation in adult mice indeed upregulate the expression of *Tbr1* and that the elevation of TBR1 can further induce *Grin2b* expression. We also found that activity-dependent *Tbr1* expression is regulated via the NMDA receptor and calcium/calmodulin-dependent protein kinase II (CaMKII)-mediated signaling, suggesting a positive feedback loop to control neuronal activity via TBR1 and NMDAR. Our study provides evidence that *Tbr1* also plays a role in the response of mature neurons to neuronal activation.

## MATERIALS AND METHODS

### ANIMALS

*Tbr1^+/-^* mice were originally provided by Dr. Robert Hevner (Department of Neurological Surgery, University of Washington, Seattle) with the permission of Dr. John Rubenstein (Department of Psychiatry, University of California at San Francisco; Hevner et al., 2001). The mice were maintained in a specific pathogen-free temperature- and humidity-controlled facility at the Institute of Molecular Biology, Academia Sinica, and backcrossed into a C57BL/6 background for over 20 generations. Wild-type C57BL/6 mice were purchased from the National Laboratory Animal Center, Taiwan. All animal experiments were conducted with the approval of the Academia Sinica Institutional Animal Care and Utilization Committee, and in strict accordance with its guidelines and those of the Council of Agriculture Guidebook for the Care and Use of Laboratory Animals.

### ANTIBODIES AND CHEMICALS

The rabbit polyclonal TBR1 antibody (TBRC) has been described previously (Hsueh et al., 2000). The mouse monoclonal MAP2 antibody was purchased from Sigma-Aldrich. The Alexa Fluor® 488- and Alexa Fluor® 555-conjugated secondary antibodies were purchased from Invitrogen. The following drugs were used: glutamate was purchased from Invitrogen; tetrodotoxin (TTX), bicuculline, 2,3-dihydroxy-6-nitro-7-sulfamoyl-benzo(f)quinoxaline (NBQX) and NMDA were obtained from Tocris Bioscience; DL-2-amino-5-phosphonovaleric acid (AP5), cyclosporin A (CsA) and KN-93 were purchased from Sigma-Aldrich.

### PRIMARY CULTURED NEURONS

The hippocampal and amygdalar neurons from postnatal day 1 (P1) mouse pups were dissociated with papain (Sigma) and grown in neurobasal medium (Invitrogen) supplemented with 2% B27 supplement (Invitrogen), 0.5 mM glutamine, and 12.5 μM glutamate. The amygdala was dissected as described in a previous study (Lesscher et al., 2008). The cortices from embryonic day 17.5 mice were trypsinized, dissociated, and cultured in neurobasal medium with 2% B27 supplement and 0.5 mM glutamine. For immunofluorescence staining, 350,000 neurons per well were plated in 12-well plates with poly-L-lysine-coated coverslips. For quantitative polymerase chain reaction (PCR) analysis, neurons were plated at a density of 1,000,000 cells/well in poly-L-lysine-coated 6-well plates. Under these conditions, the cultures were treated with drugs and subjected to subsequent experiments after 21 *days in vitro* (DIV).

### QUANTITATIVE PCR

The RNA from cultured neurons and different brain regions of adult mice was purified as described (Huang et al., 2014). The extracted RNA was then subjected to complementary DNA (cDNA) synthesis using the Transcriptor First Strand cDNA Synthesis Kit (Roche) according to the manufacturers’ instructions. The LightCycler 480 Probes Master kit (Roche) was used for the quantitative-PCR assay. The primer sets for *Tbr1* were 5′-CAAGGGAGCATCAAACAACA-3′ and 5′-GTCCTCTGTGCCATCCTCAT-3′. The primer sets for *Grin2b* were 5′-GGGTTACAACCGGTGCCTA-3′ and 5′-CTTTGCCGATGGTGAAAGAT-3′. The primer sets for c-*Fos* were 5′-GGGACAGCCTTTCCTACTACC-3′ and 5′- AGATCTGCGCAAAAGTCCTG-3′.

### IMMUNOFLUORESCENCE STAINING

Primary cultured neurons were fixed with 4% paraformaldehyde and 4% sucrose in phosphate buffered saline (PBS) at room temperature (RT) for 10 min, permeabilized with 0.1% TritonX-100 in PBS for 10 min, and blocked with 2% bovine serum albumin (BSA) and 3% horse serum (HS) in PBS for 2 h. After blocking, the neurons were incubated with anti-TBR1 antibody (4 μg/ml) and anti-MAP2 antibody (1:500) in PBS containing 2% BSA and 3% HS overnight at 4^∘^C. After washing, the cells were incubated with the corresponding secondary antibodies conjugated with Alexa Fluor® 488 and Alexa Fluor® 555 at RT for 2 h, followed by mounting in Vectashield mounting medium (Vector Laboratories). Counter stain with 4′,6-diamidino-2-phenylindole dihydrochloride (DAPI) was performed to label nuclei of neurons. The images were captured with a confocal microscope (LSM 700; Carl Zeiss) and then analyzed with the MetaMorph analysis software (MetaMorph) to quantify the TBR1 fluorescence intensity. The single-plane image was opened and the multi-wavelength cell scoring module was used to score the TBR1 fluorescence intensity of neurons. The regions of nuclei were defined by the signals of DAPI. The average intensity of nuclear TBR1 was determined to indicate the mean fluorescence intensity of TBR1. For each experiment, about thirty neurons were collected in each treatment. At least three independent experiments were carried out. The statistical analysis was performed using the pooled data from repeated experiments.

### CONDITIONED TASTE AVERSION

The behavioral paradigm was as previously described (Huang et al., 2014). Briefly, water-deprived mice at 10–16 weeks of age were trained to receive their daily water for 15 min in the experimental cages during the pre-training period. On training day (D0), mice were offered a sucrose solution as a new pleasant taste for 15 min and were subsequently intraperitoneally injected with lithium chloride (LiCl; 0.15 M, 20 μl/g of body weight) to induce aversive responses or with sodium chloride (NaCl) as a control. Two or twenty-four hours after injection, mice were sacrificed by cervical dislocation. Different brain areas were then sampled for the subsequent quantitative PCR analysis.

### STATISTICAL ANALYSIS

**FIGURE 1:**
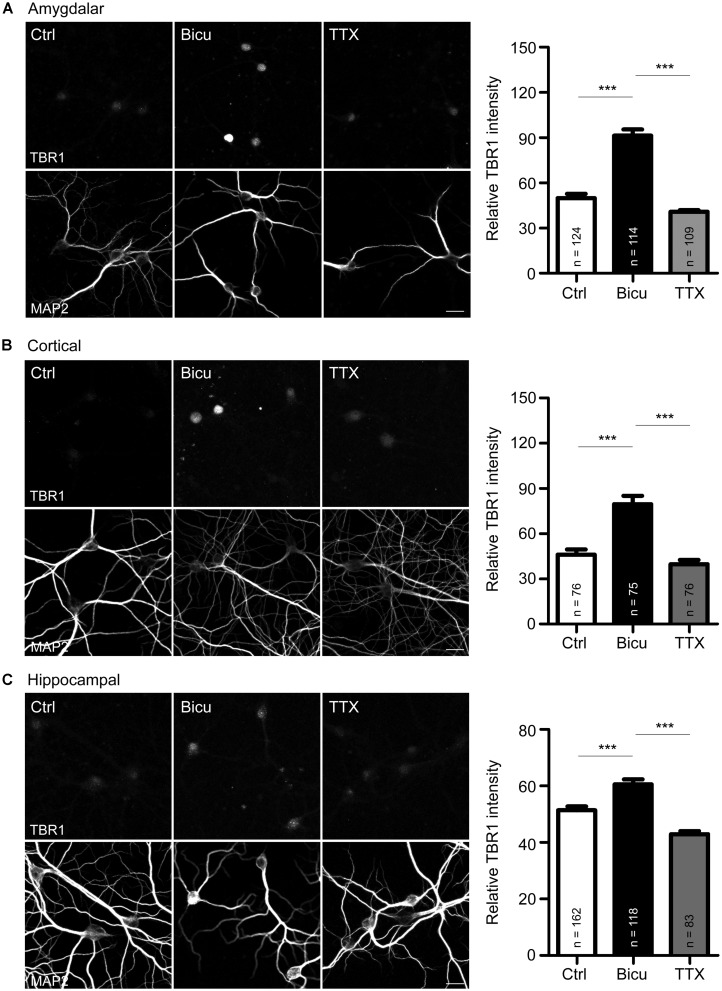
**T-brain-1 proteins are induced by neuronal activation in cultured neurons. (A–C)** Double immunostaining with TBR1 and MAP2 antibodies. Primary amygdalar **(A)**, cortical **(B)**, and hippocampal **(C)** neurons were incubated with TTX (1 μM) or bicuculline (Bicu, 40 μM) for 6 h at 21 DIV. Neurons were then fixed for immunostaining. Scale bars: 20 μm. The bar graphs in right panels of **(A–C)** show the mean fluorescence intensity of TBR1 quantified by MetaMorph software. All data are presented as the mean + SEM. The number of neurons (n) analyzed for each experiment is indicated in the figure. ***P < 0.001.

**FIGURE 2:**
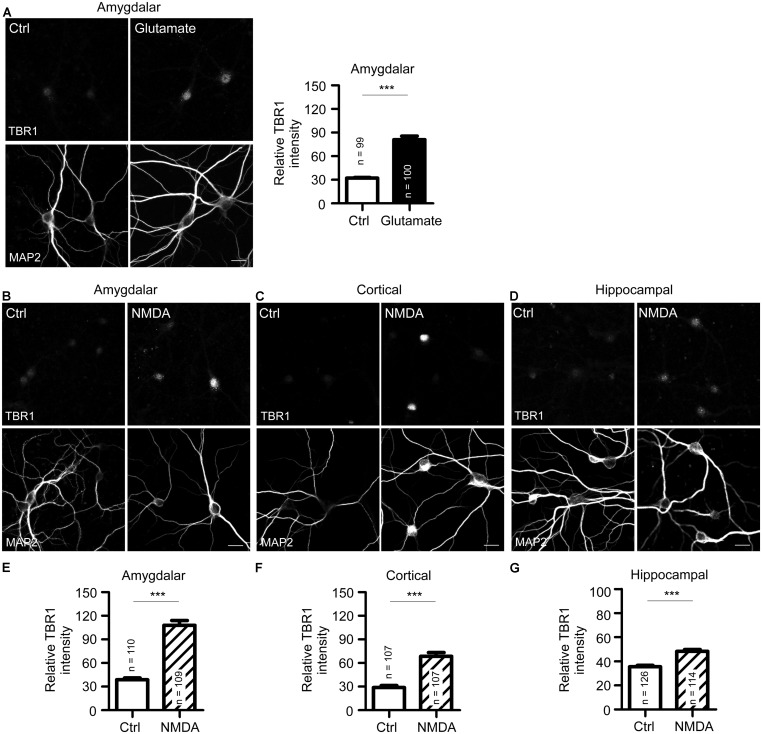
***N*-methyl-D-aspartate receptor activation induces TBR1 expression in cultured neurons. (A)** Mature amygdalar neurons at 21 DIV were treated with glutamate (50 μM) for 10 min and transferred to normal culture medium for 2 more hours. Neurons were then fixed and immunostained with TBR1 and MAP2 antibodies. Quantitative data show the relative intensity of TBR1 fluorescence. **(B–D)** The mature amygdalar **(B)**, cortical **(C)**, and hippocampal **(D)** neurons were incubated with NMDA (10 μM) for 6 h. After staining with TBR1 and MAP2 antibodies, the means of TBR1 fluorescence intensities are shown in **(E–G)**. Scale bars: 20 μm. All data represent the mean + SEM. The number of neurons (n) for each experiment is indicated in the panels. ***P < 0.001.

**FIGURE 3:**
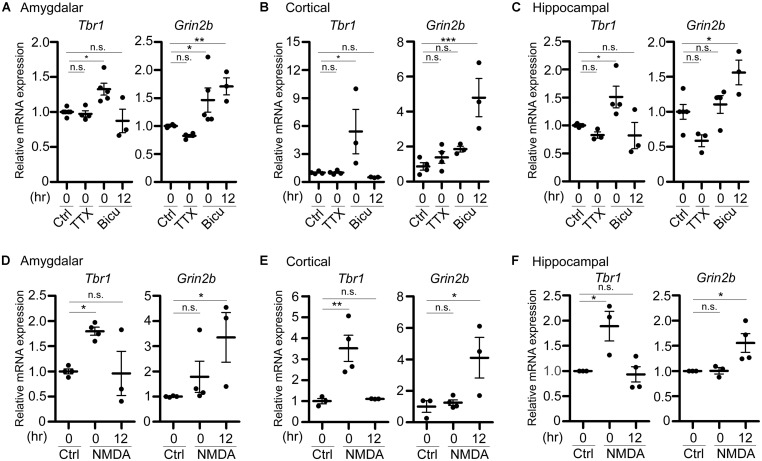
***Tbr1* upregulation correlates with *Grin2b* induction in cultured neurons.** Quantitative-PCR analyses were performed to examine the expression of *Tbr1* and *Grin2b* upon **(A–C)** TTX and bicuculline (Bicu) treatment and **(D–F)** NMDAR activation. Cultured amygdalar **(A,D)**, cortical **(B,E)**, and hippocampal **(C,F)** neurons were incubated with TTX (1 μM), bicuculline (40 μM), NMDA (10 μM), or vehicle (Ctrl) for 6 h. Immediately (0 h) or 12 more hours (12 h) after treatment, neurons were harvested for quantitative-PCR using *Cyp* as an internal control to quantify the relative mRNA levels of *Tbr1* and *Grin2b* at 21 DIV. The experiments were performed independently at least three times. Data are presented as the mean ± SEM. *P < 0.05; **P < 0.001; ***P < 0.001; n.s., not significant.

**FIGURE 4:**
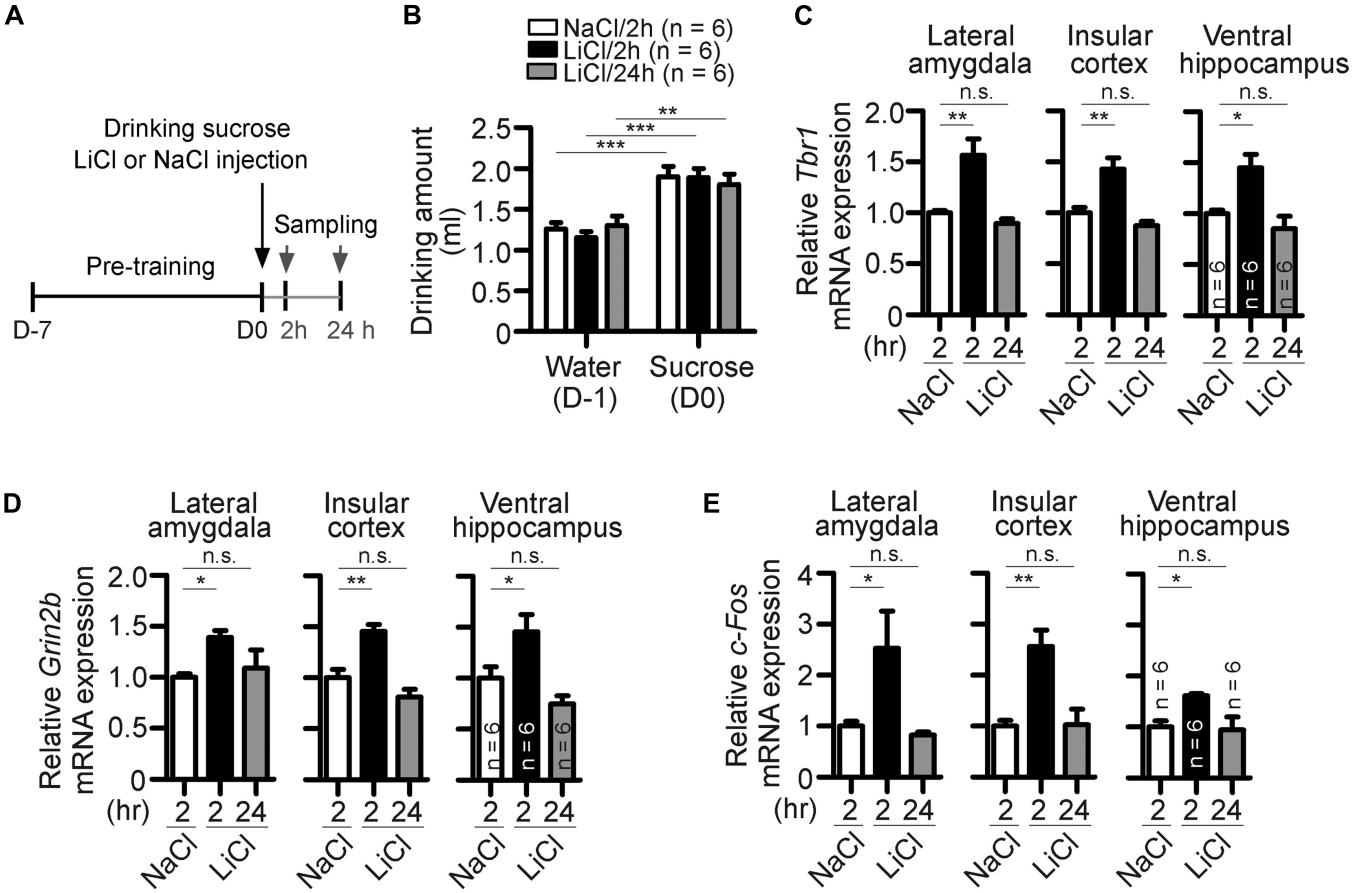
**Behavioral stimulation results in upregulation of *Tbr1* and *Grin2b* in adult brains. (A)** A simple flowchart of CTA test. The tissues were sampled 2 and 24 h after injection. **(B)** The amounts of water drunk during pre-training (D-1) and sucrose drunk on the CTA training day (D0). **(C–E)** Quantitative-PCR analyses to examine the expression of *Tbr1*, *Grin2b*, and *c-Fos* upon behavioral training. Two hours after NaCl or LiCl injection and 24 h after LiCl injection, the lateral amygdala, insular cortex and ventral hippocampus were collected for quantitative-PCR using *Cyp* as an internal control to detect the relative mRNA levels of *Tbr1* **(C)**, *Grin2b* **(D)**, and *c-Fos* **(E)** in adult mice. Six animals were used for each experiment. All data represent the mean + SEM. *P < 0.05; **P < 0.001; ***P < 0.001; n.s., not significant.

**FIGURE 5:**
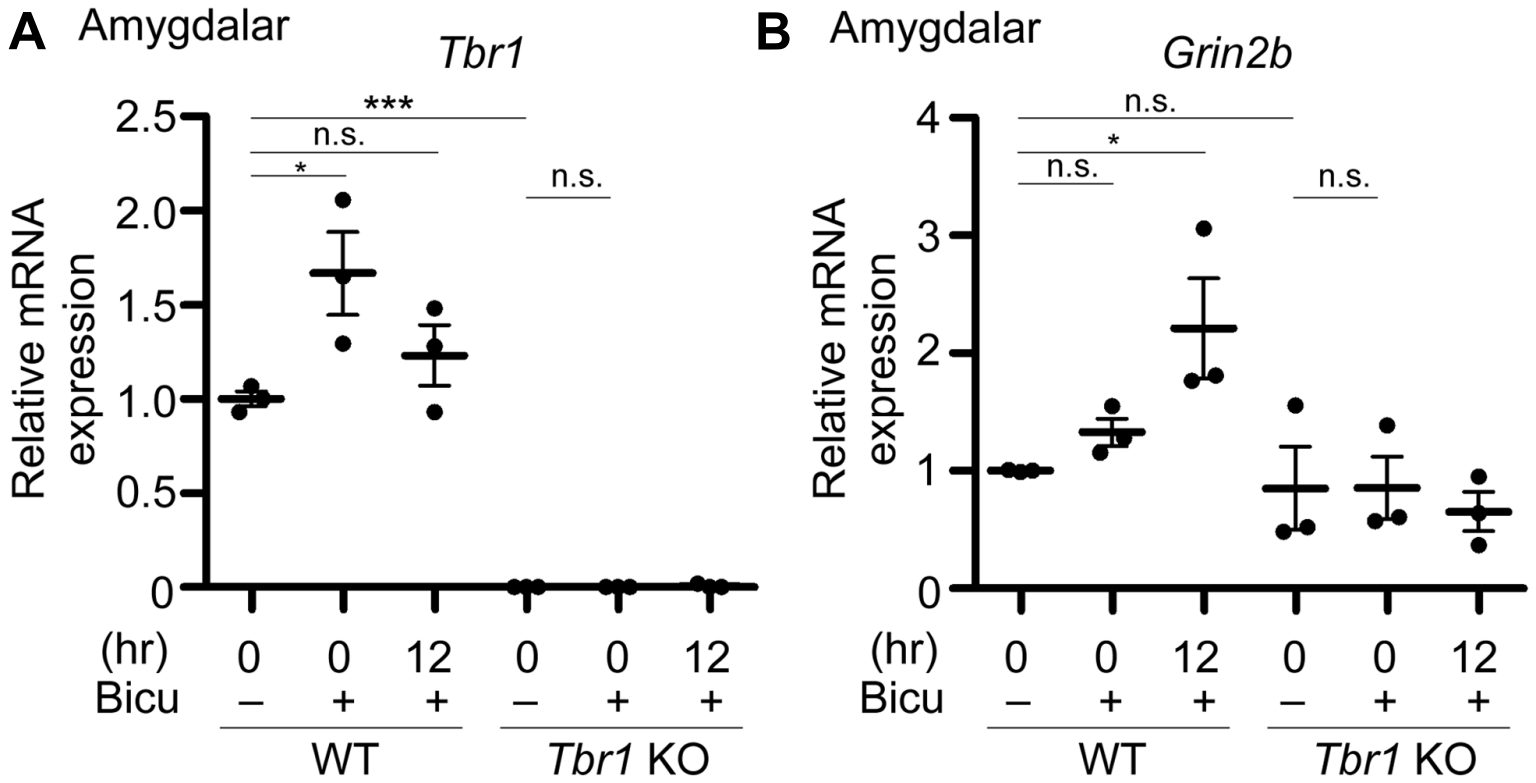
***Grin2b* induction upon neuronal activation requires *Tbr1* expression in neuronal cultures.** Bicuculline (Bicu, 40 μM) was added to the WT and *Tbr1* KO amygdalar cultures at 21 DIV for 6 h. Quantitative-PCR analysis was conducted to measure the relative mRNA levels of *Tbr1* **(A)** and *Grin2b* **(B)** immediately (0 h) and 12 h after bicuculline stimulation. The *Cyp* expression level was used as an internal control. *Tbr1* KO amygdalar neurons did not respond to bicuculline in terms of *Grin2b* induction. Experiments were repeated three independent times. Data represent the mean ± SEM. *P < 0.05; ***P < 0.001; n.s., not significant.

**FIGURE 6:**
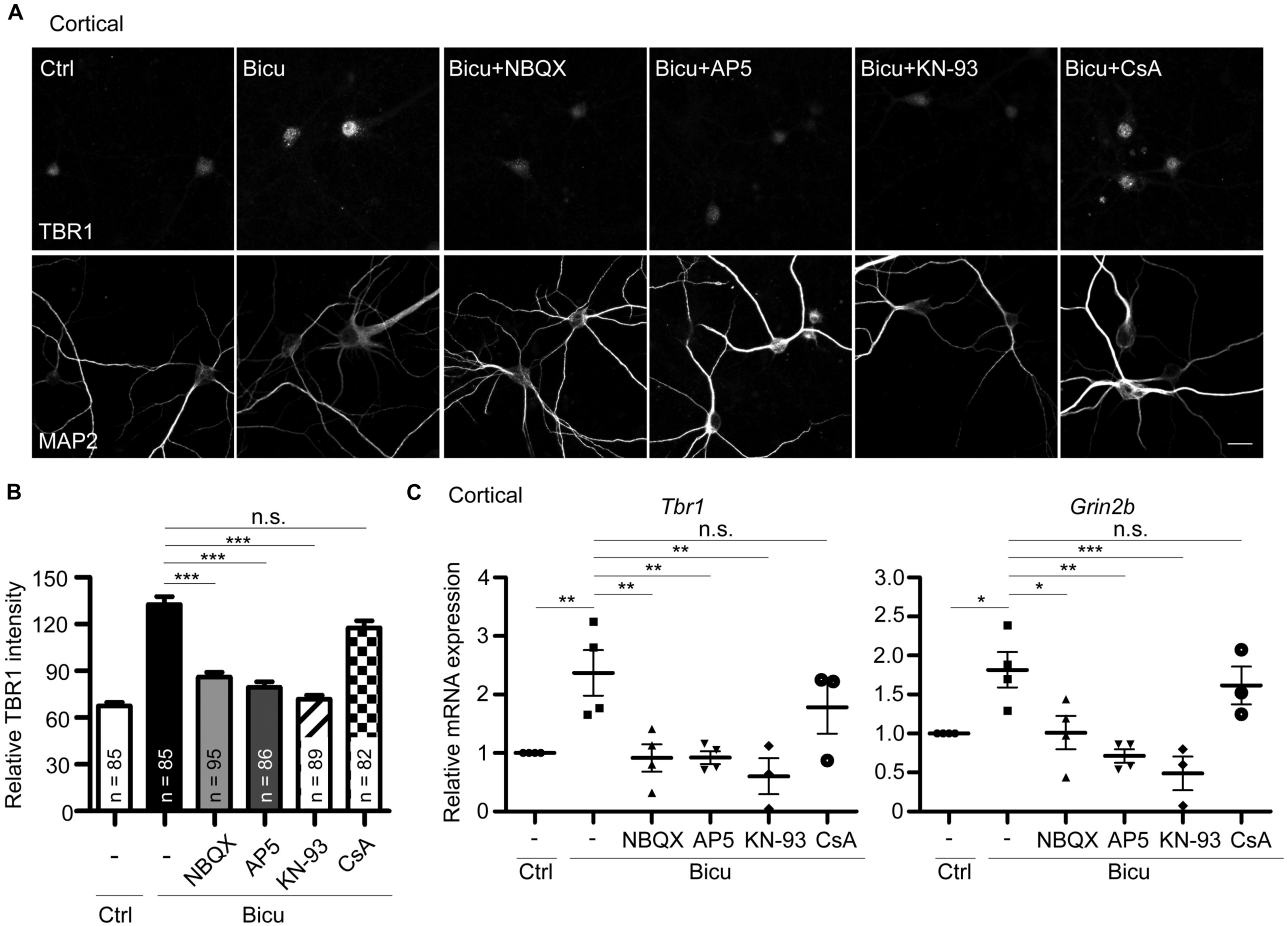
**T-brain-1 upregulation requires activation of CaMKII in neuronal cultures.** Cultured cortical neurons of WT mice were pretreated with APV (100 μM), NBQX (100 μM), KN-93 (5 μM), or CsA (5 μM) for 30 min prior to bicuculline stimulation (Bicu, 40 μM) for 6 h. Neurons were then fixed for immunostaining **(A,B)** or harvested for quantitative-PCR **(C)** at 21 DIV. **(A)** Double immunostaining with TBR1 and MAP2 antibodies. Scale bars: 20 μm. The quantitative data of TBR1 fluorescence intensities are shown in **(B)**. The number of neurons (n) for each experiment is indicated in the figures. All data represent the mean + SEM. **(C)** Real-time PCR to quantify the relative mRNA levels of *Tbr1* and *Grin2b* upon bicuculline activation. The *Cyp* expression level was used as an internal control. Experiments were repeated at least three independent times. All data represent the mean ± SEM. *P < 0.05; **P < 0.001; ***P < 0.001; n.s., not significant.

Data are presented as the mean ± SEM. The statistical analysis was performed using one-way ANOVA with a *post hoc* Dunnett test (**Figures 1, 3, 4C–E**, and **6**) and an unpaired *t*-test (**Figure 2**) in GraphPad Prism or a two-way ANOVA with a *post hoc* Bonferroni test (**Figures 4B** and **5**) via the SigmaStat software version 3.5 (Copyright^©^ 2006 Systat Software, Inc., Germany). A *P* value < 0.05 was considered significant.

## RESULTS

### THE TBR1 PROTEIN LEVELS ARE REGULATED BY NEURONAL ACTIVITY IN CULTURED NEURONS

Upon behavioral stimulation, most TBR1-positive projection neurons in the amygdalae of adult brains express c-FOS (Huang et al., 2014), a marker for neuronal activity, which suggests the involvement of TBR1-positive neurons in behavioral responses. To examine whether *Tbr1* is regulated by or participates in neuronal activation, we first examined the expression of TBR1 in mature amygdalar neurons at 21 DIV using double immunofluorescence staining with TBR1 and the neuronal marker MAP2. The cultures were incubated with the sodium channel blocker TTX (1 μm) or with the ionotropic GABA_A_-antagonist bicuculline (40 μm) for 6 h to reduce or activate neuronal activity, respectively. Consistent with the previous studies and the role of TBR1 as a transcription factor (Hsueh et al., 2000), we found that TBR1 was mainly located in cell nuclei in the absence of special treatment, though the levels were low (**Figure 1A**). Interestingly, we found that incubation with bicuculline but not TTX increased the protein expression levels of TBR1 in mature amygdalar neurons (**Figure 1A**). Because TBR1 is also expressed in the cerebral cortex and hippocampus of the adult brain (Hong and Hsueh, 2007), mature cortical and hippocampal neurons were also used to investigate the induction of TBR1 after neuronal activation. Similarly, bicuculline treatment increased the TBR1 protein levels in the cortical and hippocampal neurons (**Figures 1B,C**), though the degree of TBR1 induction in hippocampal neurons was much lower than those in amygdalar or cortical neurons (**Figure 1**). These data indicate that the protein levels of TBR1 are upregulated by neuronal activation.

### NMDAR ACTIVATION STIMULATES TBR1 EXPRESSION

Because bicuculline drives neuronal excitability by blocking the activity of inhibitory neurons and because *Tbr1* is expressed in excitatory projection neurons (Hevner et al., 2001; Huang et al., 2014), bicuculline likely reduces the inhibitory effect of inhibitory neurons and indirectly increases the expression of *Tbr1* in projection neurons. We then examined whether the direct activation of projection neurons by glutamate stimulation also increases TBR1 expression. Similar to bicuculline treatment, we found that TBR1 expression was induced in mature amygdalar neurons after glutamate stimulation (**Figure 2A**). Among the various glutamate receptors, NMDA receptors are the major glutamate receptors to induce calcium influx and play important roles in the regulation of long-term potentiation, synaptic plasticity, learning and memory. We then investigated whether the induction of TBR1 expression by glutamate depended on the NMDA receptor. NMDA treatment also effectively induced TBR1 upregulation in amygdalar, cortical, and hippocampal neurons (**Figures 2B–G**). Together, these results support that TBR1 expression undergoes NMDA receptor-dependent regulation.

### *Tbr1* UPREGULATION ASSOCIATES WITH *Grin2b* INDUCTION IN NEURONAL CULTURES

In addition to the protein levels, we performed quantitative RT-PCR analysis to examine the change of the *Tbr1* mRNA levels upon neuronal activation. Immediately after treatment with TTX or bicuculline for 6 h, cultured amygdalar neurons were either harvested for RNA extraction or washed and incubated in normal culture medium for 12 more hours. We found that while *Tbr1* expression was not affected by TTX treatment, the *Tbr1* mRNA levels were highly elevated immediately after bicuculline stimulation (labeled as “0” hour after treatment in the figure). Interestingly, we noticed that *Tbr1* expression was significantly decreased to a level comparable to that of the vehicle control at 12 h after bicuculline treatment. Similarly, the expression levels of *Tbr1* from both cortical and hippocampal neurons were upregulated immediately after bicuculline treatment and decreased 12 h after stimulation (**Figures 3B,C**), suggesting a transient induction of *Tbr1* expression after neuronal activation.

To further confirm if NMDA receptor signaling is also involved in the induction of *Tbr1* mRNA expression, we also stimulated cultured amygdalar, cortical, and hippocampal neurons with NMDA for quantitative RT-PCR analysis. We found that the mRNA levels of *Tbr1* showed a noticeable increase immediately after NMDA treatment for 6 h and declined to basal levels 12 h after NMDA washout (**Figures 3D–F**). Together with the immunostaining analysis, these data support that both the mRNA and protein levels of *Tbr1* are upregulated in an activity-dependent manner via the NMDA receptor.

Because TBR1 was shown to directly regulate the expression of *Grin2b* (Wang et al., 2004b), one of the NMDAR subunits implicated in proper neuronal activity, and because the GRIN2B expression levels were upregulated upon behavioral stimulation (Huang et al., 2014), we then assessed whether the activity-dependent upregulation of *Tbr1* expression is relevant to the induction of *Grin2b* during neuronal activation. Therefore, the RNA expression level of *Grin2b* was also examined. We found that the *Grin2b* expression levels tended to be increased immediately after treatment with bicuculline or NMDA for 6 h, although only the bicuculline treatment of amygdalar neurons resulted in significant differences from vehicle-treated neurons (**Figure 3**). In all amygdalar, cortical and hippocampal neurons, the *Grin2b* mRNA levels were noticeably upregulated 12 h after bicuculline or NMDA treatment (**Figure 3**), suggesting a time delay in the upregulation of *Grin2b* expression after neuronal activation. These data indicate that neuronal activation first induces *Tbr1* upregulation and then increases *Grin2b* expression.

### BEHAVIORAL TRAINING INDUCES *Tbr1* UPREGULATION IN ADULT MICE

We then employed behavioral stimulation to investigate whether *Tbr1* responds to neuronal activity and regulates *Grin2b* expression *in vivo*. The conditioned taste aversion (CTA) test, an amygdala-dependent learning and memory test, was chosen for behavioral stimulation. In CTA, mice will learn to correlate a novel food (sucrose) with aversive responses induced by LiCl injection (**Figure 4A**). An injection of NaCl was used as a control. Consistent with a previous observation (Huang et al., 2014), the amounts of sucrose solution drunk on the training day (D0) were higher than the amounts of water drunk during pre-training (D-1), suggesting that all mice behaved normally to prefer to drink sucrose (**Figure 4B**). Two or 24 h after training, animals were sacrificed to examine the expression of *Tbr1* and *Grin2b* mRNAs using quantitative RT-PCR. In the lateral amygdala, the expression levels of *Tbr1* or *Grin2b* were highly upregulated 2 h but significantly decreased 24 h after CTA training (**Figures 4C,D**, the groups of 2 and 24 h after LiCl injection). Because the amygdala has strong reciprocal interactions with the ventral hippocampus and insular cortex to modulate behavioral responses (Piette et al., 2012; Felix-Ortiz and Tye, 2014), we then examined whether CTA training also increases the expression levels of *Tbr1* and *Grin2b* in the ventral hippocampus and insular cortex. The induction of *Tbr1* and *Grin2b* were observed in these two brain regions (**Figures 4C,D**). These *in vivo* data support that both *Tbr1* and *Grin2b* were upregulated in the brain in response to behavioral stimulation.

Because *c-Fos* has been shown to be an IEG that is upregulated after behavioral training (Montag-Sallaz et al., 1999), we also included *c-Fos* as a positive control in the quantitative PCR assay. Similar to *Tbr1* mRNA, we found that the *c-Fos* expression levels in the lateral amygdala, insular cortex, and ventral hippocampus were upregulated at 2 h but returned to a level comparable to that of the control 24 h after CTA training (**Figure 4E**). The *c-Fos* expression pattern confirmed the neuronal activation upon behavioral stimulation in our experiment.

### TBR1 IS REQUIRED FOR THE INDUCTION OF *Grin2b* UPON NEURONAL ACTIVATION

To directly investigate whether an increase in TBR1 expression in cultured neurons is essential to *Grin2b* upregulation, *Tbr1* knockout (KO) amygdalar neurons were cultured, and their response to bicuculline was investigated. Similar to the aforementioned data, the *Tbr1* mRNA levels showed a noticeable increase in WT neurons immediately after bicuculline stimulation (**Figure 5A**). *Tbr1* mRNA was not detected in *Tbr1* KO neurons, supporting the specificity of our quantitative RT-PCR (**Figure 5A**). More importantly, we found that the *Grin2b* mRNA expression was no longer increased 12 h after bicuculline treatment in *Tbr1* KO neurons (**Figure 5B**). These results support that TBR1 is required for the induction of *Grin2b* expression upon neuronal activation.

### CaMKII IS REQUIRED FOR THE *Tbr1* INDUCTION

The above results have demonstrated the upregulation of *Tbr1* expression upon neuronal activation *in vitro* and *in vivo*. We next investigated the possible mechanisms by which neuronal activity induces *Tbr1*. Upon the excitatory synaptic transmission triggered by bicuculline or glutamate treatment, the depolarization of the plasma membrane via AMPA glutamate receptors can trigger the opening of NMDA receptors (Soderling and Derkach, 2000). Thus, we investigated whether the AMPA receptor was required for bicuculline-induced *Tbr1* upregulation. The pre-incubation of cultured cortical neurons with the AMPA receptor antagonist NBQX (100 μM; for 30 min) inhibited the induction of *Tbr1* expression by bicuculline at both the protein and mRNA levels (**Figures 6A–C**). Moreover, blocking NMDA receptor activation by incubating cells with the NMDA receptor antagonist APV (100 μM) for 30 min prior to bicuculline application noticeably inhibited activity-dependent *Tbr1* upregulation (**Figures 6A–C**), which is consistent with the above data indicating that the NMDA receptor is required for *Tbr1* induction. These data suggest that the activations of both the AMPA and NMDA receptors contribute to *Tbr1* induction following bicuculline treatment.

Because the NMDA receptor promotes activity-dependent gene expression by directly or indirectly activating a number of signaling molecules, such as CaMKII or calcium-dependent phosphatase calcineurin (Greer and Greenberg, 2008), we investigated whether CaMKII or calcineurin is required for *Tbr1* upregulation upon bicuculline treatment. To test this possibility, the mature cortical neurons were pretreated with the CaMKII antagonist KN-93 (5 μM) or calcineurin antagonist cyclosporin A (CsA; 5 μM) and then stimulated with bicuculline. Immunostaining with the TBR1 antibody indicated that pre-incubation with KN-93 could block the upregulation of TBR1 induced by bicuculline (**Figures 6A,B**), whereas CsA did not significantly affect TBR1 induction. In addition to blocking the protein levels, KN-93 but not CsA inhibited bicuculline-induced *Tbr1* mRNA expression (**Figure 6C**), suggesting that neuronal activation upregulates *Tbr1* expression in a CaMKII-dependent manner. Similar to the inhibition of *Tbr1* upregulation, we found that the *Grin2b* induction in response to bicuculline activation was also blocked by inhibiting the CaMKII activity with KN-93 (**Figure 6C**).

**FIGURE 7:**
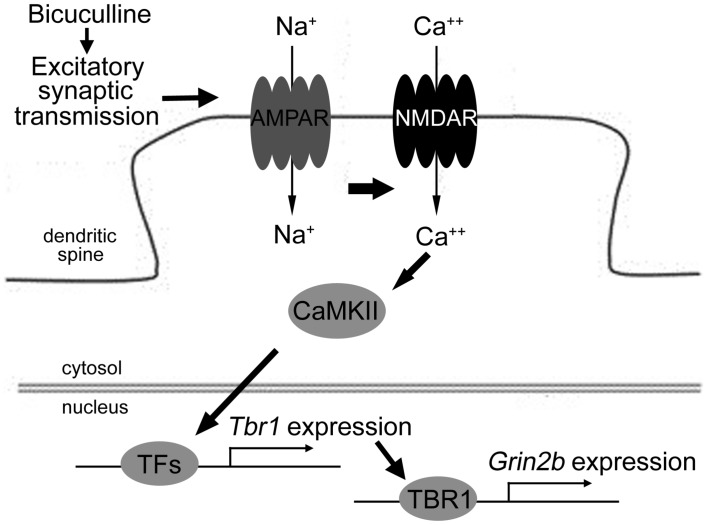
**The model of the signaling pathways elucidating *Tbr1* upregulation by neuronal activation.** Excitatory synaptic transmission driven by bicuculline treatment triggers the activation of the AMPA glutamate receptor. Na^+^ influx through the AMPA receptor depolarizes the plasma membrane and then activates the NMDA receptor. The activation of CaMKII signaling via NMDAR can induce *Tbr1* expression perhaps via other activity-dependent transcription factors (TFs). The elevation of the TBR1 protein levels can further upregulate the *Grin2b* expression following neuronal activation.

Together, these results suggest that excitatory synaptic transmission driven by bicuculline treatment can activate both the AMPA and NMDA receptors. The signaling pathway of CaMKII underlying the activation of NMDAR is involved in neuronal activation-dependent *Tbr1* upregulation. The elevation of *Tbr1* expression further increases *Grin2b* expression (**Figure 7**).

## DISCUSSION

### *Tbr1* EXPRESSION AND ITS UPSTREAM REGULATION

*Tbr1* has been shown to play a critical role in brain development during the embryonic stage (Bulfone et al., 1995, 1998; Hsueh et al., 2000; Hevner et al., 2001; Medina et al., 2004; Remedios et al., 2007; Bedogni et al., 2010; Han et al., 2011). In the current report, we provide evidence that *Tbr1* also impacts mature neurons: neuronal activation upregulates the expression of *Tbr1* to further induce *Grin2b* expression. *Tbr1* induction requires NMDA receptor-mediated excitatory synaptic transmission. Thus, TBR1 is involved in a positive feedback loop to upregulate NMDAR pathway.

Here, we show that the activation of CaMKII driven by calcium influx via NMDAR is required for *Tbr1* expression in response to neuronal activation. However, how CaMKII signaling contributes to *Tbr1* upregulation is unclear. The stimulation of NMDAR by synaptic activity activates the cyclic-AMP response element binding protein (CREB; Hardingham et al., 2002), an activity-dependent transcription factor critical for learning and memory. Furthermore, ample evidence suggests that CaMKII can regulate the rapid transcription of IEGs, including *c-Fos* and brain-derived neurotrophic factor (*Bdnf*), by phosphorylating and activating CREB (Greer and Greenberg, 2008). Phosphorylation increases the interaction of CREB with the coactivator CREB-binding protein (CBP) and induces the binding to the full palindromic CRE sequence (TGACGTCA) or the half CRE site (TGACG/CGTCA) to promote expression of target genes (Zhang et al., 2005). Using the bioinformatics database (http://natural.salk.edu/CREB/), we found the several predicted CRE sites in both human and mouse *Tbr1* genes from the range of 5 kb upstream to 1 kb downstream of the transcription start site. For mouse *Tbr1* gene, the CREB binding sites are located around the nucleotide residues -4460, -4368, -3682, -3418, -3019, +432, and +536 relative to the transcription initiation site. For human TBR1 gene, the half CRE sites are positioned around nucleotide residues -4797, -862, and +478 relative to transcription start site. These bioinformative analyses also favor that CREB is likely a candidate to induce *Tbr1* expression, although further experiments such as electrophoretic mobility shift assay and luciferase reporter assay are needed to confirm this speculation.

In addition to CaMKII, calcium influx also activates calcineurin, a calcium-activated serine phosphatase. Calcineurin plays a critical role in the synaptic plasticity through regulating the transcription factor nuclear factor of activated T cells (NFAT; Schwartz et al., 2009) and the activity of NMDA receptor channels (Lieberman and Mody, 1994; Victor et al., 1995). It also triggers nuclear export of histone deacetylases and thus modulates *c-Fos* expression (Parkes and Westbrook, 2011). To test the role of calcineurin in *Tbr1* expression, we have applied cyclosporine A to the cultures and found that inhibition of calcineurin with cyclosporin A (CsA) could not block the *Tbr1* upregulation triggered by bicuculline, suggesting that calcineurin is not involved in neuronal activity-induced *Tbr1* expression. However, in addition to regulating calcineurin, CsA has the other activities. It binds to the cyclophilins (CyPs), small intracellular regulatory proteins, to exert its immunosuppressive action. CsA is also an inhibitor for mitochondrial permeability transition by binding mitochondrial matrix-specific CyP-D and blocking its translocation to the inner membrane of mitochondria (Friberg et al., 1998). CsA treatment is also able to increase the intracellular calcium level (Kaminska et al., 2001). Failure of CsA treatment to block the *Tbr1* expression indicates that none of these pathways is involved in regulation of *Tbr1* expression.

Moreover, the expression of activity-dependent genes can be triggered by the activation of a number of signaling molecules, such as calcium-related signaling cascades, the Ras/MAPK pathway and Rac GTPases (Greer and Greenberg, 2008). Therefore, we cannot exclude the possibility of the involvement of other pathways in response to neuronal activation to induce *Tbr1* expression. Conversely, the activity-dependent epigenetic modification of the chromatin structure has been implicated in the regulation of the expression of IEGs in response to neuronal activation (Parkes and Westbrook, 2011). *Tbr1* expression is regulated by AF9/MLLT3 via the methylation of histone H3 lysine 79 at the *Tbr1* transcriptional start site (Buttner et al., 2010), indicating that epigenetic modification may be associated with *Tbr1* expression. Many studies have demonstrated that epigenetic DNA methylation at CpG dinucleotides plays important roles in the activity-dependent neural plasticity and memory formation of mature brains (Day and Sweatt, 2010; Stevenson et al., 2011). Therefore, the ability of neuronal activation-driven epigenetic regulations, such as DNA methylation, histone acetylation, or histone phosphorylation, to control the *Tbr1* expression in adult brain also constitutes an interesting avenue for future research.

Post-translational modification, such as protein phosphorylation, mediated by neuronal activity has been shown to regulate the transcriptional activity of transcription factors and thus play an important role in neuronal plasticity (Soderling and Derkach, 2000; Greer and Greenberg, 2008). Therefore, in addition to promoting the expression levels of *Tbr1*, neuronal activation may increase the transcriptional activity of TBR1 to upregulate *Grin2b* expression. In fact, our previous findings showed that the PKA phosphorylation of CASK, a co-activator of TBR1 (Hsueh et al., 2000), enhances the interaction between CASK and TBR1 and then promotes *Grin2b* expression (Huang et al., 2010). The activation of the cAMP-PKA pathway plays a critical role in learning-related synaptic plasticity and long-term memory (Kandel, 2012). Thus, the PKA phosphorylation of CASK likely indirectly increased the transcriptional activity of TBR1, which upregulated *Grin2b* expression and promoted memory formation in adult mice. In the future, exploring the cAMP-PKA pathway to elucidate the role of *Tbr1* in mature neurons will be important.

### *Tbr1* AND IMMEDIATE EARLY GENES

Our study indicated that *Tbr1* and *c-Fos* show similar transient expression patterns upon neuronal activation (Parkes and Westbrook, 2011). As discussed above, similar to *c-Fos* promoter, there are several CRE sites predicted to locate in *Tbr1* promoter. We therefore suggest that *Tbr1* may act as an IEG to modulate neural activity-dependent gene transcription. In addition to *Grin2b*, the ability of TBR1 to control the expression of other neuronal activity-dependent genes is an interesting future avenue of research.

Immediate early genes are a group of genes that are expressed at low levels in quiescent cells, but are induced transiently and rapidly at the transcriptional levels in response to a variety of cellular stimulation (Sheng and Greenberg, 1990). Many IEGs, including the *c-Fos* family, *Jun* family, *Zif268* (also termed *Egr-1*), *Nur77* and *c-Myc*, function as transcriptional factors. Some of genes encoding secreted proteins such as *Bdnf* and cytoskeletal proteins such as *Arc* comprise other important classes of IEGs (Rial Verde et al., 2006; Parkes and Westbrook, 2011). Numerous studies have demonstrated that the induction of IEGs is differentially regulated during various neural stimulation including seizure activity, stress, focal brain injury, and long-term potential, and memory formation (Hughes and Dragunow, 1995). The previous study also showed that GABAergic inhibition in primary motor cortex induces focal synchronous activation and elicits selective induction of *c-Fos* and another member of the *Jun* family, *JunB* (Berretta et al., 1997). In the report, our data also suggest the involvement of *Tbr1* in regulation of GABAergic interneurons in the IEG expression. Besides, the response of IEGs also couples to morphological plasticity. The small GTPases CDC42 and RAC1 are known to be downstream of NMDAR activation in triggering actin branching/polymerization and dendritic spine enlargement (Carlisle and Kennedy, 2005; Tolias et al., 2011; Saneyoshi and Hayashi, 2012). The actin remodeling induced by activated CDC42 and/or RAC1 is able to facilitate the association of *Arc* mRNA with remodeled actin network at the activated synapse (Huang et al., 2007). These small GTPases also influence gene expression in the nucleus. Expression of the dominant negative mutants of *Cdc42*, *Rac*, or *RhoA* impairs the activation of transcription factor serum response factor (SRF; Hao et al., 2003), which is required for neuronal activity-induced expression of IEGs such as *c-Fos*, *Arc*, and *Egr1* (Ramanan et al., 2005). It suggests that the Rho GTPases reorganize actin cytoskeleton and thus control the SRF activity to activate IEG expression (Greer and Greenberg, 2008). Another example is the expression of *pip92*, an IEG critical for neuronal differentiation. RAC1 or CDC42 regulates the expression of *pip92* through the activation of p21-activated kinase (PAK1; Park et al., 2007). Thus, the Rho GTPase family also contributes to the regulation of IEGs.

### *Tbr1* UPREGULATION AND NMDAR ACTIVITY IN AUTISM

In our analyses, *Tbr1* expression was induced after the activation of the NMDA receptor, followed by an increase in the *Grin2b* mRNA levels. Our previous studies also indicated that TBR1 directly binds the *Grin2b* promoter and controls the expression of luciferase reporter under the control of *Grin2b* promoter (Wang et al., 2004a,b; Huang et al., 2010). Taken together, *Tbr1* likely contributes to neuronal activation-dependent *Grin2b* expression via a positive feedback loop in mature neurons. Interestingly, our recent data demonstrate that *Grin2b* induction upon behavioral stimulation is impaired in *Tbr1^+/-^* mice, which thus show autism-like behaviors (Huang et al., 2014). Because the reduction of *Grin2b* likely leads to the deregulation or hypo-activation of NMDAR activity, it then results in an aberrant excitation/inhibition balance. Because the excitation/inhibition imbalance in the brain is highly associated with ASD (Rubenstein and Merzenich, 2003; Won et al., 2012), the impairment of neuronal activation in *Tbr1^+/-^* mice likely cannot trigger *Tbr1* upregulation and thus cause the lack of *Grin2b* induction. Therefore, these findings support the hypothesis that neuronal activation-dependent *Tbr1* upregulation plays critical roles in the regulation of its target genes relevant to synaptic modification, learning, memory, and psychiatric disorders. *Tbr1* has been indicated as high-confidence risk gene for ASDs (Neale et al., 2012; O’Roak et al., 2012a; Huang et al., 2014). In addition to truncated mutants, two missense mutations, N374H and K228E, had been identified in patients with ASD (Neale et al., 2012; O’Roak et al., 2012b). Our previous studies indicated that the N374H mutation impairs the ability of *Tbr1* to control the differentiation of amygdalar neurons (Huang et al., 2014). However, whether these autism-linked mutants of *Tbr1* disrupt the ability of *Tbr1* to sense neuronal activation signals and whether the N374H mutation influences the DNA binding ability or protein stability to further affect TBR1 function remains unclear. Further investigation is needed to elucidate these possibilities.

Note that we reported in an earlier study that NMDA stimulation results in a decrease of *Grin2b* in neurons (Wang et al., 2004a). Our data presented here indicate that *Grin2b* expression is upregulated after the activation of the NMDA receptor. This difference is likely due to the degree of activation of the NMDA receptor. In our previous study, 100 μM NMDA was used to stimulate neurons, while 10 μM NMDA was applied in the current study. Because low-dose NMDA leads to the phosphorylation and high-dose NMDA induces de-phosphorylation of CAMKIIα at Thr286 (Bozdagi et al., 2010), the opposite effect on CaMKII phosphorylation may then lead to different consequence in terms of *Grin2b* expression.

Another interesting data observed in this report is that only treatment with bicuculline, but not NMDA, significantly increased the expression of *Grin2b* right after 6 h stimulation. Bicuculline administration blocks the inhibitory effect of GABA_A_ receptors, one of major receptors existed in interneurons, and indirectly promotes excitatory synaptic transmission mediated mainly by glutamatergic neurotransmission (Ferraro et al., 1999; Ch’ng et al., 2012). All of ionotropic glutamate receptors (NMDAR, AMPAR, and kainate receptors), metabotropic glutamate receptors and voltage-gated calcium channels can be indirectly activated by bicuculline and lead to calcium influx (Niciu et al., 2012; Banerjee et al., 2013). However, NMDA is a specific agonist for NMDA receptor and has no action on other glutamate receptors. Our condition (10 μM NMDA for 6 h) may not be strong enough to induce *Grin2b* expression.

In conclusion, our study showed that neuronal activation increases the mRNA and protein levels of *Tbr1* via NMDAR-dependent activation. The upregulated TBR1 expression then promotes the expression of *Grin2b* in mature neurons and adult mice. This study elucidates the physiological significance of *Tbr1* in adult brains and suggests the potential involvement of the activity-dependent upregulation of *Tbr1* in cognition.

## AUTHOR CONTRIBUTIONS

Hsiu-Chun Chuang designed and performed all of the experiments, analyzed the results, and drafted the manuscript. Tzyy-Nan Huang contributed to promoter analysis and discussion. Yi-Ping Hsueh designed the experiments and drafted the manuscript. All of the authors read and approved the final version of manuscript.

## Conflict of Interest Statement

The authors declare that the research was conducted in the absence of any commercial or financial relationships that could be construed as a potential conflict of interest.
